# Disease Severity, Activity, Impact, and Control and How to Assess Them in Patients with Hereditary Angioedema

**DOI:** 10.3389/fmed.2017.00212

**Published:** 2017-12-04

**Authors:** Anette Bygum, Paula Busse, Teresa Caballero, Marcus Maurer

**Affiliations:** ^1^HAE Centre Denmark, Department of Dermatology and Allergy Centre, Odense University Hospital, Odense, Denmark; ^2^Icahn School of Medicine at Mount Sinai, New York, NY, United States; ^3^Allergy Department, Hospital La Paz Institute for Health Research (IdiPaz), CIBERER U754, Madrid, Spain; ^4^Department of Dermatology and Allergy, Charité – Universitätsmedizin Berlin, Berlin, Germany

**Keywords:** hereditary angioedema, severity, activity, impact, burden, quality of life, patient-reported outcomes, control

## Abstract

Hereditary angioedema (HAE) is a group of rare, potentially life-threatening, and frequently debilitating diseases characterized by recurrent, and often with an unpredictable onset, of swelling attacks. HAE is heterogeneous, with considerable differences between its subtypes, patients, and even within the same patient over time. During the past few years, several new on demand and prophylactic therapies have become available for HAE, allowing for individualized treatment. Therefore, to optimize HAE management, it is important to determine in all patients, the severity of their attacks, their disease activity, its therapeutic control, and its impact on their quality of life. In this manuscript, we review the existing tools to assess these aspects of HAE management, many of which are patient-reported outcome instruments. Also, we outline the current gaps of knowledge and what tools are still missing to allow for a comprehensive assessment of all patients with HAE including children.

## How Hereditary Angioedema (HAE) Affects Patients and Why Physicians Need to Assess Symptom Severity, Disease Activity, Disease Control, and Quality of Life (QoL) Impairment in Their Patients

Hereditary angioedema is a group of rare, potentially life-threatening, and debilitating diseases characterized by recurrent swelling attacks. These attacks affect the skin and mucosa including the airway, gastrointestinal tract, and genitourinary region (1). Classically, the edema in HAE patients develops gradually over several hours, increases slowly for 12–36 h, and then subsides after 2–5 days if not fatal or treated. HAE may be severely debilitating and potentially life-threatening. Patients may experience painful abdominal attacks with a very sudden and severe onset as well as laryngeal edema with acute airway obstruction. Attacks frequently occur after a trauma, and in the case of the airways, after events such as tooth extraction or tongue piercing (1).

Hereditary angioedema is a very heterogeneous disease with a fluctuating and unpredictable course (2–4). The frequency of attacks varies considerably between patients and within patients, from virtually never to twice weekly. HAE symptoms often begin in childhood and reoccur lifelong. In most families, other family members are affected, and many patients have ancestors who died of asphyxiation due to an HAE attack (5).

The classically described HAE types I and II are characterized by a deficiency of antigenic or functional complement C1 inhibitor (C1-INH) and are referred to as C1-INH-HAE (6). More recently, HAE with normal C1-INH has been described as an estrogen-sensitive variant with a higher penetrance in women (6).

The variety of clinical manifestations, together with a common unfamiliarity among physicians and emergency staff, makes diagnosis, and treatment of this disorder particularly challenging. Based on these inherent diagnostic challenges, the mean delay to diagnosis can be as long as 16 years, even in developed countries (5, 7), with a decrease to 10.3 years more recently (8). In addition, patients may have difficulties in receiving proper treatments for acute HAE attacks as they are not available in all hospitals (9).

The burden of HAE includes physical, psychological, and social effects of the recurrent, unpredictable, disfiguring, painful, and potentially life-threatening attacks (10, 11). Additional emotional stress occurs when medical professionals fail to recognize the nature and severity of HAE, and when patients are not involved in the diagnostic and therapeutic process (12). In patients with untreated or inadequately controlled disease, a significant impact on work, school, and social activities is seen. In a Danish cohort study, more than half of patients felt that HAE had a significant psychological impact on their lives and subsequently, restricted their physical activities (8). Almost two-thirds worried about the risk of suffocation, and more than one-third worried about possible side effects of HAE treatment. Patients with HAE have higher levels of anxiety and depression than the background population (10–12).

Using the generic QoL questionnaires, SF-12 and SF-36, a reduced QoL in HAE patients was shown in many studies (10, 13–16). Between attacks, patients have a significant psychosocial burden due to fear of the next painful abdominal attack or the possibility of a fatal laryngeal swelling (8, 11, 17). Many patients make long-term lifestyle modifications to accommodate their disease, with restrictions in their careers, partnerships, and recreational activities (10, 18). Furthermore, many patients have feelings of guilt about passing the disease to their children or decide not to have children (11, 19). Family members share the burden of patients, as they are often involved in the care of their relatives with HAE (20).

A conceptual model has been developed to illustrate the health-related QoL impacts of HAE (21). The model depicts five main areas of disease burden: unnecessary treatments and procedures, symptom triggers, attack impacts, caregiver impacts, and long-term impacts.

During the last 10 years, several new evidence-based and very effective treatments have become available. For the treatment of attacks, early therapy and self-administration are recommended and are now widely practiced in many countries (21). Long-term prophylaxis with intravenous plasma derived C1-INH has been introduced (22), and additional prophylactic treatment options are under development (23). An international guideline and consensus papers have been published to promote evidence-based treatments (24–26). In many countries, comprehensive care centers have been established, with HAE specialists providing patients with tailored treatment, comprehensive disease management plans, and support at the psychosocial level (8, 17, 27–29). Recent studies show that the burden of illness improves significantly in HAE patients, who have access to newer therapies, expert care, and home therapy (8, 30).

In order to individualize and optimize the treatment of HAE patients, physicians need to understand their patients (i.e., the severity and frequency of attacks, the current disease activity, the impact of the disease on patients’ lives and QoL, as well as how well the disease is controlled). Attack severity, disease severity, disease activity, impact of the disease, and disease control are conceptually different but linked. Here, we review and discuss the importance of these different aspects of HAE and present the tools that are currently available to assess them.

## HAE Disease and Attack Severity

Disease severity and disease activity, in HAE and in other chronic diseases, are different concepts. Disease severity relates to the overall disease experience of the patient. It includes the previous problems that the disease has imposed on the patient since its onset, the current burden of disease, and the long-term risks and prognosis, including the patient’s fears of potential problems to arise (31). In contrast, disease activity is the sum of the current problems (over a specified period of time) that a patient has experienced with his or her disease. Disease activity fluctuates, due to changes in the exposure to eliciting triggers and in response to treatment (32, 33). It can be precisely quantified, if appropriate tools are available, and is discussed in detail in the subsequent section. In contrast, disease severity tends to be more stable than activity, yet more difficult to classify and is discussed below.

The disease severity of HAE is difficult to classify for several reasons. First, disease activity likely alters disease severity. However, the disease activity of an individual HAE patient is highly variable over time, and, therefore, may not necessarily reflect the severity of HAE at a given time point (34). The availability of more tolerable and effective medications to prevent attacks and to treat attacks early after onset, likely alters disease activity and consequently HAE severity. Furthermore, classifying HAE disease severity is based upon subjective findings regarding a patient’s perception of the impact of disease on his or her QoL and on daily activities. To one patient, hand swellings may be perceived as insignificant, whereas to another, severe, as they impose major limitations in daily activities. Importantly, HAE is a disease in which all patients are at risk for an airway attack and death by asphyxiation, regardless of past symptoms, which makes it difficult to classify a disease as “mild.” Consequently, HAE is felt to be a severe disease in all patients given that all patients are at risk of developing laryngeal attacks and suffocation, even those without previous laryngeal attacks. Finally, validated biomarkers that reliably measure disease severity are not available. As a result, creating an instrument for assessing HAE severity is difficult.

Tools to measure HAE disease severity have been proposed (19, 35–37). These tools vary in regards to which factors are addressed, including the age of symptom onset, sex, and attack locations, whether the patient used prophylactic therapy, duration and frequency of attacks, and the perceived impairment on daily activities. However, none of the reported tools to measure HAE severity have been validated, nor do they address the specific needs of pediatric or adolescent patients. They also vary in the recall period. In 2011, Bygum and coworkers reported the use of a clinical HAE severity score, based on the age at disease onset, the number of organs ever affected, and the need for long-term prophylaxis, with values from 0 to 10 (37). This score is not commonly applied in clinical practice. Another score was developed at the 2003 C1-Esterase Inhibitor Deficiency Workshop (36). This score includes the numbers of attacks in the past year (with each attack quantified by its impact on the patient’s ability to perform daily activities) and the need for treatment over the past year including emergency treatments and the use of long-term prophylactic therapy. Although reported as a severity measure, this score, by and large, describes disease activity during the past year, with low- and high-cumulative values corresponding to low- and high-disease activity, respectively. In summary, HAE severity is hard to classify and, therefore, the development of validated tools remains a challenge.

In contrast, the assessment of the severity of a specific HAE attack is straightforward and several tools have been created and used. HAE guidelines recommend that patients and their providers monitor attack severity and attack frequency (6, 25, 28). Tools for quantifying attack severity are critical for clinical trials to quantify treatment effects on HAE attacks. Patient-reported outcome (PROs) measures such as the visual analog scales (VAS) have been used in trials for acute HAE therapy, and some of these scales have undergone content validation (38–40). VAS scores, although easy to compute, typically assess a single sign or symptom, and do not provide a composite score, yet would likely be able to be used by patients for their monitoring of an attack as part of their self-care.

Two additional measures have been developed to provide a composite score (41). The Mean Symptom Complex Severity (MSCS) is a global score incorporating the numbers of anatomical sites effected during an acute attack, termed the “symptoms complex” (e.g., hands and abdomen) and the patients’ assessment of the severity of swelling at each site (e.g., none to severe). It measures the severity of an attack at a specified time point, such as at baseline, prior to administration of a study medication, or a time period after drug administration. The Treatment Outcome Score (TOS) is comprised of three components, the “symptoms complex,” the baseline severity, and the response at 4 and 24 h post-dosing. This score provides a numeric value to assess a patient’s response to therapy. Although the MSCS and the TOS are validated and address multiple anatomical locations of angioedema during a specific attack, they are somewhat complex to calculate and not likely to be used for clinical practice. They have also been used for one medication, Ecallantide, and as a result, the scores cannot be used to compare it to other HAE medications not assessed by the TOS or MSCS. Taken together, PRO measures for assessing attack severity and frequency are available and valuable tools, as they allow for a standardized and reproducible documentation of HAE attacks over time. Attack severity should be assessed in routine clinical practice and in clinical trials.

## How to Measure Disease Activity and Control in HAE Patients

Patient-reported outcome tools for assessing HAE activity are used to determine how active the disease is at a given point in time. Two validated PRO tools are readily available, the angioedema activity score (AAS), and the hereditary angioedema activity score (HAE-AS) (42, 43).

The AAS is the first symptom-specific PRO measurement developed to assess angioedema activity. It is validated for all forms of recurrent angioedema including HAE. The AAS is a diary in which patients prospectively document on a daily basis, the presence or absence of angioedema during the past 24 h. If angioedema was present, patients answer five additional questions, each scored from 0 to 3 points. Thus, the minimum and maximum daily score can be between 0 and 15 points, respectively. Cumulative data from four consecutive weeks, labeled AAS28, provides a valid assessment of disease activity (42). The AAS has a one-dimensional structure, excellent internal consistency, good levels of validity, and test–retest reliability, and it is sensitive to changes of angioedema activity over time. Its minimal important difference is 8 points for the 7-day cumulative AAS (AAS7).

The HAE-AS is a specific PRO tool designed to assess disease activity in C1-INH-HAE (43). It was developed following psychometric and Rasch analysis and documents retrospective disease activity in the last 6 months. It consists of 12 items, which show unidimensionality. The raw score (0–29) is transformed into a linear measure with a scale from 0 to 30. This linear measure shows good internal consistency (Cronbach’s alpha 0.81), satisfactory reliability for group comparisons (Person separation index = 0.748), and good discriminative validity by age, gender, and disease severity (*p* < 0.05).

The AAS and the HAE-AS are similar in some aspects, yet different in others (see Table 1). Both tools are available free of charge to physicians for the use in routine clinical practice (AAS: www.moxie-gmbh.de; HAE-AS: www.idipaz.es). The AAS has been used in randomized controlled trials to assess angioedema activity (32, 44). Comparative studies that use both the AAS and HAE-AS are needed to better understand and compare the results obtained with these PRO tools.

**Table 1:** Characteristics and availability of relevant patient-reported outcome measures for disease activity and QoL impairment in HAE.

	HAE-AS	AAS	HAE-QoL	AE-QoL
Number of items (questions)	12 (once)	1–6 (everyday)	25 (once)	17 (once)
Recall period	6 months	1 day	6 months	4 weeks
Applicable in HAE 1/2	+	+	+	+
Applicable in other forms of recurrent angioedema	−	+	−	+
Assessment	Retrospective	Prospective	Retrospective	Retrospective
High level of patient compliance required	−	+	−	−
Clinical important difference published	−	+	−	+
Cost-free use in routine patient management and investigator-initiated clinical research	+	+	+	+
Different language versions available^a^	American-English, Spanish	American-English, Azeri, Canadian-English, Canadian-French, Danish, Dutch, French, German, Greek, Hebrew, Hungarian, Italian, Japanese, Macedonian, Mexican-Spanish, Polish, Portuguese, Romanian, Russian, Slovakian, Spanish, Swedish, Turkish	Argentinien-Spanish, Austrian-German, Brazilian-Portuguese, Canadian-English, Canadian-French, Danish, French, German, Greek, Hebrew, Hungarian, Italian, Macedonian Mandarin-Chinese, Polish, Romanian, Spanish, US-English, UK-English	American-English, Azeri, Canadian-English, Canadian-French, Danish, Dutch, French, German, Greek, Hebrew, Hungarian, Italian, Japanese, Jordan-Arabic, Macedonian, Mexican-Spanish, Polish, Portuguese, Puerto Rican-Spanish, Romanian, Russian, Slovakian, Spanish, Swedish, Turkish

*AAS, angioedema activity score; AE-QoL, angioedema quality of life questionnaire; HAE-AS, hereditary angioedema activity score; HAE-QoL, hereditary angioedema quality of life questionnaire*.

*^a^Additional language versions of the HAE-AS and HAE-QoL (check www.idipaz.es for updates) and the AAS and AE-QoL (check www.moxie-gmbh.de for updates) are in preparation*.

Disease control is an important concept in chronic diseases, especially those characterized by recurrent attacks and fluctuating disease activity. Tools that assess disease control, determine the level of control that patients have of their disease at a specific time point. Examples of PRO measures that assess disease control are the asthma control test and the urticaria control test (45–48). Control tests can also be retrospective PRO measures and usually have defined cut off values that indicate poorly controlled disease. They are especially useful for assessing patient responses to preventive/prophylactic therapy, as they are quick to complete, can be used at every patient visit, and results are instantly available after completion. The development and validation of an angioedema control test is currently underway.

## QoL Impairment in HAE

Health-related quality of life (HRQoL) is the individual perception of the impact of a disease, disability, or symptom across physical, psychological, social, and somatic domains of functioning and well-being (49). Impaired HRQoL in patients with HAE has been documented by using generic instruments (e.g., SF-12, SF-36, and EQ-5D) (10, 14–16, 18, 50) and dermatology-specific questionnaires (DLQI) (51). Generic instruments (E5QD) have been used to estimate health status utilities (18, 32). Generic instruments (SF-12 and SF-36), dermatology instruments (DLQI), and pain instruments (pain disability index) have also been used to assess the effect an intervention has on HRQoL of patients with C1-INH-HAE (7, 52–54). Bouillet et al. found that HRQoL measured by SF-36 in patients with HAE was lower than in patients with allergy in France (*p* < 0.05) (14). More studies comparing HAE with other frequent and known diseases are needed. It is important to note that none of the generic PROs has been validated for its use in HAE.

Whereas generic questionnaires are useful for HRQoL comparison among different diseases, they often have a low sensitivity to assess particular facets of a disease (55). Therefore, condition-specific questionnaires are usually preferable when evaluating the impact of individual diseases and their treatment on HRQoL impairment. Specific instruments, AE-QoL and HAE-QoL, are available and have been validated for angioedema and HAE, respectively (19, 56–58). One additional tool, the HAEA-QoL, is currently under development in the United States.

The AE-QoL is a symptom-specific questionnaire, which assesses HRQoL in patients older than 18 years with any type of recurrent angioedema (57). It consists of 17 items, which address four dimensions: functioning, fatigue/mood, fears/shame, and food. It has a recall period of 4 weeks. Its psychometric properties are good, with total internal consistency being 0.89 and test–retest reliability 0.83. It shows a total score value and four domain score values which vary from 0 to 100 after a linear transformation of raw values. Higher score values indicate a lower HRQoL (57). AE-QoL is also sensitive to change, with a minimal clinical important difference of six points (58, 59). The AE-QoL has been used in some randomized clinical trials in bradykinin-mediated and mast cell mediator-mediated angioedema (32, 44, 59–61) and in clinical practice (59). Finally, AE-QoL is the recommended tool for assessing QoL in urticaria patients with angioedema in the EAACI/GA^2^LEN/EDF/WAO guideline for urticaria (62–64).

The HAE-QoL is a specific questionnaire for adult patients with C1-INH-HAE. It consists of 25 items grouped into seven HRQoL domains: treatment difficulties, physical functioning and health, disease-related stigma, emotional role and social functioning, concern about offspring, perceived control over illness, and mental health (19). It has been developed following a qualitative methodology, based on a patient-centered perspective (19, 56). It has a 6-month recall period. The HAE-QoL showed good internal consistency (Crombach’s alpha 0.92) and test–retest reliability (intraclass correlation coefficient 0.87), as well as a good discriminant validity in the psychometric analysis (19). HAE-QoL has been used in clinical practice (65).

A summary of the validated PROs available used in HAE is shown in Table 1. Comparative studies between AE-QoL and HAE-QoL are needed to better understand differential properties of both PROs. Differences in the recall period and the aim of the assessment (treatment response, patient clinical follow-up) should be taken into account when choosing the most appropriate PRO.

There is no validated specific PRO for the use in children with angioedema. As of now, there are only three published studies in which HRQoL was assessed in children with C1-INH-HAE. One used the Kidscreen-27 to study HRQoL in children from a big family with HAE in Colombia (50). The other used the Pediatric Quality of Life Inventory (PedsQL™) 4.0 Generic Core Scales (child self-report and maternal proxy report) to assess HRQoL in children with HAE from Hungary and Israel (66), while the third used PedsQL and the childrens DLQI (67).

Normalization of HRQoL should be a goal in patient care and treatment. HAE experts recommend that HRQoL be measured annually (6) and the World Allergy Organization C1-INH-HAE guidelines state that HRQoL should be considered when assessing the need for maintenance treatment (25). In order to better quantify HRQoL in patients with C1-INH-HAE, its determinants and the effects that therapeutical interventions have on it, data on large populations are needed.

## Gaps of Knowledge, Unmet Needs, and Conclusion

Hereditary angioedema is a variable and burdensome disease. All patients should be assessed for their disease activity, the frequency and severity of attacks, the impact of the disease on their daily lives, and their QoL, as well as the control of their disease at every visit. Today, validated tools are available for these measurements, with the exception of disease control, for which a specific tool is currently under development. Some of the tools available have been translated to several languages and culturally adapted, but further work is needed to make all tools available for all patients in all countries. Also, none of the available tools have been adapted or validated for their use in children and adolescents. The development of PRO tools for children and adolescents with HAE, thus, remains an imminent unmet need. Also, many of the tools available, although developed in line with FDA and EMA requirements and recommendations, have yet to receive official approval by these regulatory authorities.

## Author Contributions

All authors have contributed to the conception, development, drafting, and finalization of the manuscript.

## Conflict of Interest Statement

PB: funding for clinical trials from CSL/Behring, Shire, Dyax, and BioCryst. AB has been involved in clinical research and educational events involving CSL-Behring, Jerini AG/Shire, Sobi, Dyax, and BioCryst. TC has been involved in clinical trials/clinical studies/registries, has been advisor, has been speaker and/or has received educational grants for BioCryst, CSL-Behring, Dyax, GSK, MSD, Novartis, Pharming Group NV, Shire HGT, and SOBI. MM: speaker and/or advisor and/or recipient of institutional funding of research/clinical trials: BioCryst, Dyax, Moxie, and Shire.

## Abbreviations

AAS, angioedema activity score; C1-INH, complement C1 inhibitor; HAE, hereditary angioedema; PRO, patient-reported outcome; QoL, quality of life.
